# Perceived Parent-Adolescent Relationship, Perceived Parental Online Behaviors and Pathological Internet Use among Adolescents: Gender-Specific Differences

**DOI:** 10.1371/journal.pone.0075642

**Published:** 2013-09-30

**Authors:** Qin-Xue Liu, Xiao-Yi Fang, Zong-Kui Zhou, Jin-Tao Zhang, Lin-Yuan Deng

**Affiliations:** 1 Key Laboratory of Adolescent Cyberpsychology and Behavior, Ministry of Education, Wuhan, China; 2 School of Psychology, Central China Normal University, Wuhan, China; 3 Institute of Developmental Psychology, Beijing Normal University, Beijing, China; 4 National Key Laboratory for Cognitive Neuroscience and Learning, Beijing Normal University, Beijing, China; 5 Faculty of Education, Beijing Normal University, Beijing, China; Universidad de Granada, Spain

## Abstract

This study examined the associations between adolescents’ perceived relationships with their parents, perceived parental online behaviors, and Pathological Internet Use (PIU) among adolescents. Additional testing was carried out to determine the effect of different genders (parent and adolescent). Cross-sectional data was collected from 4,559 students aged 12 to 21 years in the cities of Beijing and Jinan, People’s Republic of China. Participants responded to an anonymous questionnaire concerning their Internet use behavior, perceived parental Internet use behaviors, and perceived parent-adolescent relationship. Hierarchical linear regressions controlling for adolescents’ age were conducted. Results showed different effects of parent and adolescent gender on perceived parent-adolescent relationship and parent Internet use behavior, as well as some other gender-specific associations. Perceived father-adolescent relationship was the most protective factor against adolescent PIU with perceived maternal Internet use positively predicting PIU for both male and female adolescents. However, perceived paternal Internet use behaviors positively predicted only female adolescent PIU. Results indicated a different effect pathway for fathers and mothers on boys and girls, leading to discussion of the implications for prevention and intervention.

## Introduction

As central socializing agents for children, parents provide emotional connections, behavioral constraints, and modeling, which affect children’s development of self-regulation, emotional expressions, and expectations regarding behavior and relationships [1,2]. Theories about deviance and problem behavior have thus also included parents as central elements in their explanation framework. Social control theory, one of the most influential theories of deviance, proposes that parental bonding with children inhibits deviance or problem behavior by making youth aware of the costs and effects of such behavior on their ties with others [3]. According to this theory, strong bonds with parents, usually measured as the strength of the ties of affection between parents and children, serve as a protective factor for adolescent deviance, regardless of the parents’ involvement in deviant behavior. Social learning theory, however, emphasizes the modeling effect of parents’ behaviors and parental reinforcement of children’s negative behavior [4]. Empirically, research has found that good relationships with parents may protect youth from initiating risky behavior [5,6], while observing risk-taking by parents may endorse these behaviors and increase the likelihood that their children will adopt these behaviors [7,8]. Regardless of the focus on these studies, it is clear that both the parent-child relationship and parental behavior are associated with adolescent problem behavior.

Due to the different roles fathers and mothers play in most families, researchers have paid increasing attention to the gender differences in parents’ influences on adolescent behaviors and development. Studies have shown that mothers are more likely to be seen as more effective, involved and significant than fathers in the context of family relationships [9,10]. However, a growing number of researchers have been emphasizing fathers’ contribution to the cognitive and emotional development of children and adolescents [11,12]. In these studies, the quality of the father-child, rather than mother-child, relationship operated as a strong predictor of adolescents’ school adaptation, coping strategies and social interaction, especially in relation to their anxiety and withdrawal behavior [12]. At the same time, studies have demonstrated that perceived relationships with mothers as relatively more intimate and engaged, and involved higher levels of conflict, than relationships with fathers [13,14]. In sum, these studies suggest that the quality of adolescents’ relationships with their fathers and mothers occupy independent working models and influence different domains of child development.

Nowadays, the online world has become an essential part of many adolescents’ lives. In China, using the Internet has become one of the most popular leisure-time activities among adolescents, a finding common in other countries [15-17]. Internet-related risky behavior among adolescents has been simultaneously attracting increasing parental attention, as many parents are concerned that Internet use may lead their children to become isolated from others, expose them to sexual or violent images, displace more worthwhile activities, and risk their privacy [18]. Furthermore, studies have found that adolescents had a high risk for loss of control in their online activity and develop symptoms of pathological Internet use (PIU) [19,20], also referred to in the literature as Internet addiction [21], problematic Internet use [22], compulsive Internet use [23], and Internet dependence [24]. The key features of Pathological Internet Use (PIU) include excessive or compulsive preoccupation with and loss of control over Internet use [22]. According to studies around the world, PIU is one of the most common problem behaviors for adolescents, with a prevalence rate higher than 10% in some places [16,17,25]. In China, 6% to 11% of over 200 million adolescent Internet users may pathologically use the Internet [26-28]. Previous research found that fathers and mothers reacted differently to adolescent Internet use. For example, mothers had a better awareness of adolescent Internet use than fathers [29], but fathers tended to play a more active role compared to mothers [30]. Wang et al. [31] also found that fathers were more likely than mothers to check their children’s browser history. Lei and Wu [15] have consistently found a significant association between adolescents’ perception of paternal attachment and adolescent PIU, with alienation as a positive predictor and trust as a negative predictor. Other research has suggested that fathers and mothers have comparable effects as both over-intervention and strict punishment by fathers and rejection and lack of warmth from mothers were related to adolescent PIU [32]. These studies further support our belief that the associations between fathers, mothers and adolescent PIU depend on gender specific, independent working models.

Consistent with results from studies on other problem behaviors (e.g., smoking, drug use, pathological video-gaming), perceived parent-adolescent relationship was found to be related to PIU among adolescents and served as a potential protective factor [33,34]. Parents’ Internet use behavior has received little attention as the majority of adults use Internet for work purposes, which is considered normal and necessary rather than a problematic behavior that may affect children negatively. However, recent research has indicated that parental Internet use is a model for adolescent behavior and affects adolescents’ evaluations of the advantages and disadvantages of particular behaviors [16]. It seems that adolescents whose parents use the Internet frequently could be more likely to use the Internet excessively. A recent study showed that when parental norms were consistent with their Internet use behaviors, parental norms negatively predicted adolescent PIU; conversely, when parental norms were inconsistent with their Internet use behaviors, parental behaviors positively predicted adolescent PIU [28]. These studies suggest that perceived parental Internet use behaviors may be more influential than parental norms. Thus both perceived parent-adolescent relationship and parental behavior should be examined for their associations with adolescent Internet use. Accordingly, situated in the theoretical framework of social control theory and social learning theory, the present study aimed to test the associations between adolescent PIU and adolescents’ perception of their relationship with parents and parental behavior. Based on the literature review above, we proposed the following hypotheses:

Hypothesis 1: Adolescents’ perceived relationship with their parents will negatively predict adolescent PIU, perceived parental Internet use behavior will positively predict adolescent PIU.

Furthermore, evidence has suggested that the gender of the parent and child differentially effect children’s behavior. For example, a study showed that initiation of smoking behavior among girls, when compared with boys, was more influenced by parental smoking and perceived parental approval of smoking [35]. For girls, not having any smoking parents was associated with a longer duration of abstinence [36]. However, another study found that parental smoking behavior had a stronger effect on boys’ smoking behavior than girls’ [37]. And the similar conflicting results were found in related problem behavior area which also focused on the interaction effect of parent and adolescent gender. A study found that mothers were more encouraging of risk taking by boys in comparison to girls and were slower to intervene once the risk behavior escalated to a point that posed an even greater risk of injury [38]. Based on cross-sectional data from a national sample of 1308 tenth graders, Luk et al. [39] found the association of mother and father communication with adolescent substance use varied by substance and gender. Among sons, perceived communication with father was protective against marijuana use while perceived communication with mother was protective against smoking. For daughters, neither communication with father nor mother was protective against substance use. Furthermore, researchers have found gender matching between parental variables and adolescent behaviors. For example, Patock-Peckham and Morgan-Lopez [40] found that when a parent’s gender is the same as the child, permissive parenting was directly related to impulsivity, a significant mediator of parenting effects on children’s control of alcohol consumption. Similarly, the perception of having an authoritarian father was positively associated with higher levels of neuroticism in sons only [41]. There is little research, however, on the interaction effects of parent and child gender in the Internet use literature. Li & Zhou [42] found that parent monitoring negatively predicted PIU for both boys and girls, while parental ignoring and material awarding positively predicted PIU for girls. Both Van den Eijnden et al. [16] and Liu et al. [28] reported associations between adolescent PIU and perception of parental relationship among adolescents with compulsive Internet use, but did not analyze the possible effects of gender. However, research focused on gender differences in other similar problem behaviors pointed to the importance of understanding the gender-specific differences in correlates of adolescent behaviors and perception of parent-adolescent relationship for prevention and intervention purposes [36,39]. The second purpose of the study, therefore, was to show how the associations between perception of parent-adolescent relationship and parental Internet use and adolescent PIU varied according to both parent and adolescent gender. We proposed the following hypothesis:

Hypothesis 2: The associations between adolescent perceived relationship with parents and parental Internet use behaviors and adolescent PIU will differ according to the gender of both parents and adolescents. Perceived relationships with fathers may predict both male and female adolescent PIU and perceived maternal behavior may only predict male adolescent PIU.

## Methods

### Sample

Adolescents (N=4,877) were recruited from 11 junior high schools and 12 senior high schools in the cities of Beijing and Jinan. All schools and classes were randomly selected for this study. Of this group, 318 students were excluded because they did not complete the entire questionnaire, or did not provide data on variables involved in the study. Because the present study aimed to explore the gender differences of perceived relationships with fathers and mothers among adolescents, adolescents with single parents (N=149) or other caregivers (N=5) were also excluded. This resulted in the inclusion of a total of 4,559 students (2,211 boys and 2,348 girls), with an average age of 15 years (SD =1.94), in this study. Among this group, 68.3% of students’ fathers and 64.5% of student’s mothers had attained high school and above educational levels, while 3.8% of fathers and 6.3% of mothers had educational levels lower than primary school.

### Measures and procedures

This study was approved by the Institutional Review Board of the Institute of developmental Psychology, Beijing Normal University. The assessment was conducted in school classrooms and participants were invited to complete the questionnaire anonymously after informed consent was obtained from their teachers. Parental or guardian informed consent was also obtained in this study. These forms were sent and received by the schools themselves and were also kept by the schools’ teaching offices. After receiving permission from parents concerning the study, the school’s teachers then signed our informed consent forms. Oral informed consent was received from all participants who were free to refuse without consequences participation in our study. This process, including data collection, was carried out by trained graduate students and who effectively explained and emphasized to all participants the authenticity, independence and integrality of all answers, as well as confidentiality about information collected. During the data collection process, participants filled out measures regarding their demographic information (e.g., age, gender, and grade), family income, pathological Internet use, perceived parental Internet use behavior, and parent-adolescent relationship.

### Pathological Internet use

The Adolescent Pathological Internet Use Scale (APIUS) developed by Lei and Yang [26] was used to measure Pathological Internet Use. The APIUS contained 38 items with 6 domains: salience (e.g., I forget nearly everything else when I am online), tolerance (e.g., I find that I spend more and more time on line), withdrawal symptoms (e.g., I feel upset when I cannot access the Internet), mood alteration (e.g., Going online makes me feel better when I am depressed), social comfort (e.g., I feel safer when communicating with others through the Internet) and negative outcomes (e.g., I have some difficulty with school performance because I spend too much time on the Internet). Respondents were asked to rate the extent to which each item was true for them on a five-point scale (1 = never true to 5 = always true). High scores indicated high PIU involvement. Participants with average scores higher than 3.15 were considered to have PIU, a determination based on previous studies [15,43]. The APIUS has been used widely in China, and its internal consistency coefficients have been reported in previous studies to be greater than 0.95 [15,28,43]. The Cronbach’s alpha in this study was 0.96.

### Adolescents’ perception of parent-adolescent relationship

Closeness to Parents Scale [44] was used to assess adolescent perceived father-adolescent and mother-adolescent relationships. It contained nine items and each item included father and mother as two categories. The following questions were asked about each parent using a scale from 1(not at all) to 5 (very): How close do you feel to your [mother/father]? How openly do you talk with your [mother/father]? How careful do you feel you have to be about what you say to your [mother/father]? How comfortable do you feel admitting doubts and fears to your [mother/father]? How interested is your [mother/father] in talking to you when you want to talk? How often does your [mother/father] express affection or liking for you? How well does your [mother/father] know what you are really like? How confident are you that your [mother/father] would help you if you had a problem? If you needed money, how comfortable would you be asking your [mother/father] for it? How interested is your [mother/father] in the things you do? The Cronbach’s alphas in the study were 0.91 (father) and 0.91 (mother).

### Perception of parental Internet use behavior

This study developed its own questionnaire, based on the dimensions of CNNIC’s questionnaire [45], to measure adolescent perception of parental Internet use behavior. This questionnaire included five items for five different online activities: browsing information and news, playing online games, chatting online, trading online and entertainment online (movie or music). It measured the frequency of perceived Internet use of fathers and mothers on a 5-point scale (1 = never to 5 = always). The average score of the father subscale and mother subscale was used to represent the average level of perceived parental Internet use in this study. The Cronbach’s alphas in the study were 0.78 (father) and 0.79 (mother).

### Data analysis

After data-cleaning procedures, the data were analyzed by frequency and MANOVA was used to explore the gender differences in perceived parental factors. After correlating the scores of adolescents’ perceived parent-adolescent relationship, perceived parental Internet use and PIU, two separate hierarchical multiple regressions controlled for adolescents’ age were conducted for female and male adolescents to explore the different roles of the father-adolescent, mother-adolescent relationship, and paternal and maternal Internet use in regards to their effects on adolescent PIU. All statistical analyses were conducted using the Statistical Package for the Social Science (SPSS), version 16.0.

## Results

### Adolescent pathological Internet use

According to the cutoff point of APIUS, 10.8% of participants (N = 518) were identified with PIU. The prevalence of PIU in different subgroups and results of ANOVA test of PIU in demographic factors are presented in Table 1. The table illustrates that 13.5% of boys and 8.2% of girls had PIU problems, with a significant difference in the rates of PIU between genders (χ^2^= 31.77, p < 0.001), indicating that male students had a higher possibility to have PIU problems than female students.

**Table 1:** Means and Rates of PIU in Male and Female Adolescents.

		Average on PIU	Rate of PIU
		M(SD)	N(%)
Overall		2.14(.83)	518(10.8)
Gender	Male	2.26(.84)	271(13.5)
	Female	2.03(.80)	169(8.2)
		F= 95.14^***^	χ^2^=31.77^***^

^***^ p<.001.

### Gender differences in perceived parental Internet use and parent-adolescent relationship

Results of gender differences of perceived parental Internet use and parent-adolescent relationship are presented in Table 2. Significant differences were found between male and female adolescents in most tested variables with the exception of perceived paternal Internet use. Female adolescents perceived more maternal Internet use, better father-adolescent relationship and mother-adolescent relationship than males. To explore the gender-specific differences of perceived parental factors, paired sample t-test analyses were conducted for female and male adolescents. Perceived paternal Internet use was higher compared to maternal Internet use and mother-adolescent relationships were better than father-adolescent relationships for both male and female adolescents.

**Table 2:** Gender Differences of Perceived Parental Internet Use and Parent-adolescent Relationships.

		Paternal Internet use	Maternal Internet use		F-A relationship	M-A relationship	
		M(SD)	M(SD)	t	M(SD)	M(SD)	t
Overall		1.85(.69)	1.79(.70)	6.29^***^	3.24(0.97)	3.40(0.95)	-18.78^***^
Gender	Male	1.83(.70)	1.76(.69)	4.79^***^	3.21(0.99)	3.32(0.97)	-9.65^***^
	Female	1.87(.67)	1.81(.69)	4.01^***^	3.28(0.95)	3.48(0.93)	-16.37^***^
	F	4.03^*^	5.11^*^		5.31^*^	33.62^***^	

Note: F-A, father-adolescent; M-A, mother-adolescent.

^*^ p<.05, ^**^p<.01, ^***^p<.001.

### Gender differences in the associations between perceived parent-adolescent relationships, perceived parental behavior and adolescent PIU

The results of correlating analyses and two separate multiple hierarchical regressions on male and female adolescents are shown in Tables 3 and 4. Using correlating analyses, all perceived parental factors were found to be significantly related to adolescent PIU. Multiple hierarchical regressions showed the differential impact of fathers and mothers on adolescent PIU. On the whole, perceived behavior and relationship with fathers were more influential than mothers for adolescent PIU. For both genders, adolescents’ age, perceived father-adolescent relationship and maternal Internet use were predictive of adolescent PIU. Perceived paternal Internet use positively predicted PIU in females but not males. However, perceived mother-adolescent relationship did not predict PIU for either females or males.

**Table 3:** Correlating Analysis of Perceived Parental Variables and Adolescents’ PIU.

	1	2	3	4	5
1.Paternal Internet use	-				
2.Maternal Internet use	.56^**^	-			
3.F-A relationship	.14^**^	.05^*^	-		
4.M-A relationship	.10^*^	.12^**^	.80^**^	-	
5.adolescent PIU	.13^**^	.16^**^	-.21^**^	-.20^**^	-

^*^ p<.05, ^**^p<.01, ^***^p<.001.

**Table 4:** Summary of Hierarchical Multiple Regressions for Perceived Parental Internet Use, Parent-adolescent Relationship and PIU.

		Male adolescents(N=2211)					Female adolescents(N=2348)				
		B	SE	*β*	*F*	*ΔR^2^*	B	SE	*β*	*F*	*ΔR^2^*
Step	1				55.40^***^	.03				42.61^***^	.02
	Age	.07	.01	.16^***^			.04	.01	.12^***^		
Step	2				36.58^***^	.06				45.21^***^	.09
	Age	.06	.01	.14^***^			.04	.01	.12^***^		
	Paternal Internet use	.06	.03	.05			.09	.03	.08^**^		
	Maternal Internet use	.11	.03	.10^***^			.08	.03	.08^**^		
	F-A relationship	-.11	.03	-.14^***^			-.15	.02	-.20^***^		
	M-A relationship	-.04	.03	-.04			-.04	.03	-.06		

Note: F-A, father-adolescent; M-A, mother-adolescent.

^*^ p<.05, ^**^p<.01, ^***^p<.001.

## Discussion

This study explored a differential effect of perception of parent-adolescent relationship and parental Internet use on adolescent PIU utilizing a large sample of high school students. The prevalence of PIU in this study was 10.8% overall, 13.5% for boys and 8.5% for girls. These estimates were a little bit higher than those reported in some surveys in China but were consistent with survey results in other countries. For example, a prevalence rate of 7.4% was reported for Beijing [26] and 6.8% nationwide [46], while research from western countries and South Korea have reported rates higher than 10% and even up to 18.3% [16,17,25]. These different results can be partly attributed to differences in measurements and timing, and in China’s case in particular at least, partly to the negative effects of the rapid development of the Internet in China. In the last five years alone, Chinese adolescent Internet users have increased from 100 to 250 million [47]. Exponentially increasing numbers of adolescents can access the Internet and are easily swept up by the increasing regular use of the Internet in society as a whole.

It is worth noting that adolescents’ perception of parent-adolescent relationship and parental Internet use differed according to the gender of both adolescents and parents. Firstly, perceived parent-adolescent relationship and parental Internet use differed by adolescents’ gender, with female adolescents more likely to perceive more maternal Internet use, better father-adolescent and mother-adolescent relationships than males. These results indicated that female adolescents tended to be more aware of parental behaviors and parent-adolescent interactions. Compared to boys, girls were believed to have closer relationship with their mothers [48] and experience higher levels of parental knowledge, parental control and solicitation, and disclosure [49]. Secondly, adolescent perception of parent also differed by parents’ gender. Adolescents reported less Internet use and better relationships with mothers than father. These results were consistent with previous research that adolescent reported closer and more engaged relationships with mothers than fathers [13], as well as confiding more in mothers than fathers [50]. Speaking with fathers was generally more difficult for girls than boys, and mothers were more involved in parenting adolescents than fathers, which made it easier for both girls and boys to talk to and develop better relationships with their mothers [51].

Augmenting these results, this study revealed that the associations of perceived parent-adolescent relationship and parental behavior on adolescent PIU differed by both parent and adolescent gender, partially proving our hypotheses. Adolescents’ perception of higher levels of Internet use by mother and lower levels of father-adolescent relationship predicted a greater possibility of adolescent PIU. As perceptions of father-adolescent relationships and fathers’ behavior both and mothers’ behavior only were found to predict to adolescent PIU, father seems to have more predictive power for adolescent PIU than mother. Results also showed a different effect pattern for male and female adolescents. Among boys, perceived relationship with father negatively predicted PIU and perceived maternal Internet use positively predicted PIU. For girls, however, perceptions of both paternal and maternal Internet use were predictive of PIU, while perceived relationship with father also negatively predicted PIU. In sum, on the behavioral level, girls’ PIU was influenced by perceptions of both paternal and maternal Internet use while boys’ PIU was more sensitive to mothers than fathers. On the relationship level, perceived father-adolescent relationship had greater influence on adolescent’s PIU than mother-adolescent relationship for both genders. In other words, Internet use behavior of both fathers and mothers influenced girls’ PIU and perceived relationship with father could protect both girls and boys from PIU. Findings from other problem behaviors support the results as, for instance, girls were found to be more influenced by parental smoking than boys [35] and perceived parental gambling behavior had a predictive power for female adolescents while perceived peer gambling behavior was found to be a predictor for only male adolescents [52]. Additionally, it is possible that surfing the Internet is considered more ‘normal’ behavior for boys than girls, giving boys a greater tendency for PIU. Overall, although there are gender differences, these results are consistent with social learning theory as parental behavior clearly affects via modeling adolescent PIU.

The current study also revealed that adolescents’ perceived father-adolescent relationship, and not perceived mother-adolescent relationship, predicted adolescent PIU even the average score of adolescent perceived mother-adolescent relationship was higher than that of perceived father-adolescent relationship. This is possibly due to the following reasons: Firstly, mothers in China pay significant amounts of attention to their adolescent children, including their behavior, emotions and states of mind, such that Chinese mothers are more involved with their children than their fathers, and even mothers in the United States [53]. It is thus possible that regardless of adolescent Internet use, most adolescents reported a relatively high mother-adolescent relationship, Secondly, these results are consistent with previous research that have shown that attachment to fathers was significantly associated with adolescent PIU [15]. As emphasized by Attachment Theory, strong parent-child bonds are crucial for the development of children’s ability to explore the greater outside world. If parents are unsupportive or insensitive, children may use manipulative strategies to obtain attention from their parents; when they move into a different social environment, such as a peer group cybercommunity, these manipulative coping strategies may prove maladaptive [6]. As fathers also provide important, playful interaction that give their children the opportunity to test out and develop important social skills, adolescents who feel alienated from their fathers may lack necessary social skills and appropriate coping strategies for real-world interactions. These children may more easily form online relationships where social cues can be removed or recreated to develop feelings of intimacy and closeness. These characteristics lead such children to be more vulnerable to developing into pathological Internet users [15]. Thirdly, these results may be partially explained by Chinese cultural characteristics. For example, in China, traditional and still influential notions of fathers as dominant, but distant heads of the family could exacerbate adolescents’ vulnerability for developing PIU as adolescents who feel alienated from their fathers could be more likely to feel rejected and seek connection in the cyber world.

This study’s results conflict with gender role theory that suggests boys would be more heavily influenced than girls by their relationship with their fathers. Studies focused on gender differences have also found results conflicting with gender role theory. For instance, Coley et al. [54] found that father knowledge and family activities were more protective for girls than boys regarding risky sexual behaviors. Review articles focused on the associations between fathers’ involvement and children’s developmental outcomes have also indicated that father’s engagement seems to have differential effects on desirable outcomes by reducing the frequency of behavioral problems in boys and psychological problems in girls [55]. Apparently, gender differences in the associations between paternal behavior and adolescent behavioral problems can differ by domain. Clearly, why parental Internet use affects adolescent PIU differ by gender and how this may change over the course of different development stages are important questions worthy of further research.

The results of this study may also have important implications for prevention and intervention efforts in PIU, particularly in light of the different role of perceived relationship with and behavior of fathers and mothers for adolescents. Previous studies have pointed to the association between family factors and adolescent PIU [33,56], with some scholars additionally highlighting that potential protective effect of parent-adolescent relationship on adolescent PIU [8,34]. Our findings have shown that both parent and adolescent gender differentially affect the associations between perceived parental factors and adolescent PIU, a finding with important implications for both parents and clinicians. Parents need to know and understand that their behaviors may incite or encourage their children’s Internet use; mothers who use the Internet at home need to pay attention to the increased possibility of their sons developing PIU. Most importantly, fathers are encouraged to realize the effect of the father-adolescent relationship on adolescents and become more engaged with their children to protect them from PIU. Fathers need to pay particular attention to the way they interact with their adolescent children, for what adolescents perceive in their relationship significantly affects their behavior and choices. Additionally, children’s genders need to be taken into consideration when parents choose parenting practices. For clinicians, family factors, especially parental factors, must be given due attention as well; checking parental Internet use behavior as well as adolescents’ relationships with their fathers could be especially useful when working with adolescents with PIU.

This study does have certain limitations that necessitate a cautious interpretation of the results. First, the cross-sectional nature of this study limits our ability to draw directional or causal conclusions. Second, the lack of data regarding parents limits our picture of parent-adolescent relationships and parental behavior. Future studies on adolescent PIU and parental factors should include parental perceptions of their own behaviors and interactions with their children in addition to adolescents’ perceptions of their parents’ behaviors and interactions. Bi-directionality of these associations in a family has been encouraged to be explored in future studies [16]. A better understanding of the dynamic correlations between adolescent PIU and parental factors will be useful in forming effective intervention for PIU. Longitudinal studies are also needed to adequately examine the direction of the effects.
